# Enhancing wound healing through deep reinforcement learning for optimal therapeutics

**DOI:** 10.1098/rsos.240228

**Published:** 2024-07-31

**Authors:** Fan Lu, Ksenia Zlobina, Nicholas A. Rondoni, Sam Teymoori, Marcella Gomez

**Affiliations:** ^1^ Applied Mathematics, Baskin School of Engineering, University of California, Santa Cruz, CA, USA

**Keywords:** deep learning, reinforcement learning, optimal adaptive control, wound healing, optimal treatment regime

## Abstract

Finding the optimal treatment strategy to accelerate wound healing is of utmost importance, but it presents a formidable challenge owing to the intrinsic nonlinear nature of the process. We propose an adaptive closed-loop control framework that incorporates deep learning, optimal control and reinforcement learning to accelerate wound healing. By adaptively learning a linear representation of nonlinear wound healing dynamics using deep learning and interactively training a deep reinforcement learning agent for tracking the optimal signal derived from this representation without the need for intricate mathematical modelling, our approach has not only successfully reduced the wound healing time by 45.56% compared to the one without any treatment, but also demonstrates the advantages of offering a safer and more economical treatment strategy. The proposed methodology showcases a significant potential for expediting wound healing by effectively integrating perception, predictive modelling and optimal adaptive control, eliminating the need for intricate mathematical models.

## 1. Introduction

Personalized precision treatments have become an emerging research topic in modern medicine owing to the recent advances in artificial intelligence [1–3]. The necessity of precision treatment arises from the fact that different patients exhibit different responses to a given medication. These variations stem from the molecular disparities among different patients and within the same patient at different times [4]. Personalized treatment aims to determine personalized dosages of drugs, drug types and the optimal timing of drug delivery for each patient according to current and predicted patient responses based on experimental data and statistical analysis [5]. In this article, we focus on developing an online adaptive controller using deep learning and reinforcement learning (RL) based on the patient’s real-time response to the administered treatments. This controller has been designed to expedite wound healing, but its applicability extends to other nonlinear dynamics.

Wound healing is a dynamic and continuous process that can unfold through a series of overlapping stages: haemostasis, inflammation, proliferation and maturation [6]. The process involves nonlinear transformations of different cells (platelets, neutrophils, macrophages, myofibroblasts, fibroblasts, keratinocytes and others) and biomolecules (blood coagulation factors, pro- and anti-inflammatory cytokines, polymers and enzymes of extracellular matrix) [7].

Determining the optimal timing and precise dosage for administering each drug presents a challenge, particularly when considering the different nonlinear dynamics of drug digestion and biological transformations targeted by the drug. The mechanism involved in drug distribution can be elucidated using mathematical models [8,9]. However, the complexity of biological systems and disparities within organisms reduces the reliability of model-based controllers.

Even if the model is accurate, the inherent nonlinearity further complicates the task of establishing the optimality and safety of the prescribed control policy for drug administration. This emphasizes the need to formulate controller design strategies that furnish optimal and adaptable control solutions while considering the individualized requirements of patients with specific health conditions, all under the guidance of analytically optimal and safe solutions.

Several closed-loop control strategies, such as model predictive control, optimal control and adaptive disturbance rejection control, have been suggested to control drug administration [8,10–12]. The control strategies currently in use for regulating patient drug dosing have focused on optimal drug infusion with respect to given performance measures or adaptive drug infusion that addresses patient parameter uncertainty. The main advantage of adaptive controllers is that they can derive patient-specific infusion profiles even without an accurate patient model. However, such controllers may not account for certain desired performance constraints. On the other hand, optimal controllers are predicated on nominal patient models, leading to suboptimal performance or even instability of the closed-loop system in the face of drug titration for actual patients.

The challenge here is to design an optimal treatment that accounts for gender, age, weight, pharmacokinetic and pharmacodynamic intrapatient and interpatient variability, and health conditions of the patient under treatment. In contrast to standard controller design methods, RL-based approaches allow the development of control algorithms that can be used in real time to affect optimal and adaptive drug dosing in the presence of pharmacokinetic and pharmacodynamic patient variability. The method presented in this article can be used to derive patient-specific treatment profiles, such as generating a desired patient drug response without requiring an accurate patient model. Specifically, we use a learning-based controller design strategy that can facilitate patient-specific and optimal drug titration.

Learning-based control strategies have found applications in various medical settings, enhancing the precision of drug dosing and optimizing treatment regimens. These applications include devising dynamic treatment plans for lung cancer patients [13], optimizing erythropoietin dosing during haemodialysis [14], facilitating cytotoxin delivery during chemotherapy [15], aiding insulin regulation for diabetic individuals [16] and administering anaesthetic drugs to maintain desired sedation levels [17]. Recent studies, such as those discussed in Moore *et al.* and Padmanabhan *et al.* [17,18], have delved into clinical and computational trials employing RL to enhance the precision of anaesthetic drug infusion.

However, it is worth noting that these approaches do not account for safety exploration in RL. Owing to the inherent non-convexity of objective functions and the complexity of deep neural networks, achieving a globally optimal control policy is not always assured. In the study by Padmanabhan *et al.* [19], the utilization of RL to inform drug dosing is suggested. Nevertheless, this approach still relies on prior knowledge of reference signals.

**Figure 1.**
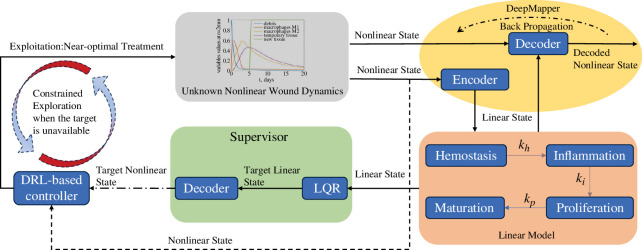
DRL-based closed-loop control to accelerate wound healing pipeline. The pipeline consists of five major blocks for performing the real-world wound state estimation in nonlinear dynamics, finding a linear representation of wound dynamics, calculating optimal reference signal in the learned linear model, supervision of the real-world wound target state and constraint exploration and exploitation of DRL agents.

Compared to the studies by Zhao *et al.*, Martín-Guerrero *et al.*, Padmanabhan *et al.*, Daskalaki *et al.* and Moore *et al.* [13–17,19], the proposed approach in this article presents a distinct advantage by formulating a leader–follower paradigm solved by deep reinforcement learning (DRL) which has demonstrated success in Zhou *et al.* [20]. We begin by learning a mapping from nonlinear wound dynamics to its linear representation. From this linear model, the optimal control law is derived, allowing the calculation of the subsequent optimal linear state. This linear state is then used by the decoder in the DeepMapper, as illustrated in figure 1, to predict the next optimal nonlinear state. This prediction serves as a reference signal for the RL agent, guiding the formulation of a treatment strategy, such as real-time drug dosages, aimed at closely matching the actual next nonlinear state to this reference state. Thanks to DRL, the regime eliminates the need for modelling the nonlinear wound dynamics or the treatment effects within it.

Learning linear representations of nonlinear systems is crucial owing to the fact that nonlinear systems are prevalent in nature, with most systems of practical interest exhibiting nonlinear behaviour, and the control of such systems is challenging with no general and scalable solution [21–23]. On the other hand, the study of linear systems is well developed with scalable design, analysis, control and optimization of linear systems thoroughly detailed within the literature [24,25].

Finding the mapping between nonlinear systems and linear models is challenging. The Koopman operator theory, as explored in seminal works in Koopman, Mezić & Banaszuk and Mezić [26–28], offers a promising avenue by enabling the representation of a nonlinear system as an infinite-dimensional linear system. However, the optimization of this approach primarily operates within the realm of functional space, rendering it often intractable in practical applications. Moreover, it does not account for the effect of control inputs in nonlinear systems.

In recent years, many advances have been made to generalize the Koopman operator theory for the control of nonlinear systems [21,29–31]. These extensions find linear representations of nonlinear systems with finite-dimensional function approximations, which can subsequently be used for the tractable control of the system. This motivated us to design a deep neural network-based algorithm called DeepMapper to learn a mapping from an unknown nonlinear system to its linear representation, similar to the goal of the Koopman Operator. The work is primarily motivated by Kaiser *et al.* and Ahmed *et al.* [30,31]. The major difference is that neither addresses the issue of overfitting during learning, which is mitigated through DRL in this article.

The main contributions of this article include the following. (i) We propose an adaptive closed-loop control framework using deep learning, optimal control and RL to enhance wound healing, as schematized in figure 1. This framework eliminates the need for mathematical modelling of nonlinear dynamics or the treatment effects within it. (ii) We propose an autoencoder-like mechanism called DeepMapper to learn a linear representation of the nonlinear wound healing dynamics, which provides an optimal reference signal for the DRL agent to track. It is shown that this regime not only improves the precision of the linear representation in modelling the wound dynamics under optimal treatments but also ensures the efficiency of the DRL agent. (iii) The experimental results show that our approach has successfully reduced the wound healing time by 45.56% compared with the one without any treatment, as well as outperforming the one with DRL directly optimized over a nonlinear system without DeepMapper. The proposed framework showcases the significant potential for expediting wound healing by effectively integrating optimal control and data-driven methods. By leveraging advanced algorithms that adapt in real time to changing conditions, this system offers a more accurate and reliable means of promoting faster recovery without relying on the limitations of conventional models.

The remainder of this article is organized as follows: §2 presents an overview of the closed-loop control framework, followed by the detailed design of the deep learning-based algorithm for finding the linear representation for nonlinear wound healing dynamics, as well as a DRL-based algorithm for accelerating wound healing. Implementation details, simulation results and a detailed discussion of these results are given in §§3 and 4. Finally, in §5, we present conclusions and future research directions.

## 2. Approach

Nonlinear dynamics characterize the vast majority of systems of practical interest. One example of this is the wound healing dynamics depicted in this article. The control and optimization of such systems, particularly in scenarios like devising the most effective wound treatment strategy, is challenging owing to their inherently nonlinear behaviour.

Conventional methods, such as linearizing around a fixed point, frequently fall short when applied to complex systems with nonlinear behaviours. These methods assume that the system’s behaviour near the fixed point can be approximated by a linear model, which is not always effective for systems exhibiting significant nonlinearity, multiple equilibria or chaotic dynamics. Consequently, they require alternative approaches that can accurately model and predict the behaviour of such systems across a broader range of conditions, bypassing the limitations inherent to linearization techniques. Alternative methods, such as machine learning and deep learning, can capture the intricate nature of these systems more efficiently through data.

For this reason, we propose a deep learning framework called DeepMapper to learn a mapping from a nonlinear wound healing dynamics to its linear representation. We show that the learned linear model can be used to provide a near-optimal reference signal to a RL controller, which will, in turn, refine the DeepMapper. This controller learns the best treatment strategy for wound care without heavily relying on mathematical interpretation of the treatment into any dynamic model. The learning framework is schematized in figure 1.

### 2.1. DeepMapper: linearization of nonlinear wound healing dynamics

Consider a nonlinear wound healing dynamical system with treatment inputs defined by:


(2.1) $$\frac{dx}{dt}=f\left(x\right)+Bu,$$

where $x\in {\mathbb{R}}^{d_{x}}$ , $B\in {\mathbb{R}}^{d_{x}\times d_{u}}$ , $u\in {\mathbb{R}}^{d_{u}}$ and $f:{\mathbb{R}}^{d_{x}}\to {\mathbb{R}}^{d_{x}}$ .

As discussed in Zlobina *et al.* [9], the function f can be an unmanageable nonlinear function that defines different cell transitions during wound healing. The nonlinear state x associated with wound healing surveyed in the literature may include variables such as pH, temperature [32–37] or visual representations captured through images of the wound [38]. We assume that x can be measured by some sensor, but f is unknown to the control algorithm.

Solving for the optimal control input $u^{⋆}$ is often difficult, particularly when the dynamics evolve nonlinearly. As extensive research and literature have been dedicated to the study of linear systems, encompassing scalable design, analysis, control and optimization [24,39], we propose the utilization of a deep learning approach to model a linear system that best approximates the behaviour of the underlying nonlinear system.

Note that equation (2.1) defines a control-affine system. As discussed in Kaiser *et al.* [30], the decoupling of the states and inputs allows us to find a transformation of the states alone:


(2.2) z=h(x),

where $x\in {\mathbb{R}}^{d_{x}}$ is the state that evolves subject to nonlinear dynamics, $z\in {\mathbb{R}}^{d_{z}}$ evolves linearly and $h:{\mathbb{R}}^{d_{x}}\to {\mathbb{R}}^{d_{z}}$ is a function that maps the nonlinear state x to linear state z .

From the chain rule, the relationship between the new state z and the original state x can be defined:


(2.3) $$\frac{dz}{dt}=\frac{dz}{dx}\frac{dx}{dt}=J_{h}\left(x\right)\frac{dx}{dt},$$

where $J_{h}(x)\in {\mathbb{R}}^{d_{z}\times d_{x}}$ is the Jacobian matrix of h .

We first seek to find a linear representation of equation (2.1) without control u satisfying the condition that the dynamics of the new state z are linear in z :


(2.4) $$\frac{dz}{dt}=Az,$$

where $A\in {\mathbb{R}}^{d_{z}\times d_{z}}$

By expanding equation (2.3) through substitution of equation (2.1) with u=0 for the time derivative term and accounting for equation (2.4) that we want to satisfy, we have


$$\begin{array}{c} \frac{dz}{dt}=J_{h}\left(x\right)f\left(x\right)\\ =Az. \end{array}$$

Then by plugging the control input u into equation (2.1) and following the same procedure, we get a linear representation with control:


(2.5) $$\begin{array}{c} \frac{dz}{dt}=J_{h}\left(x\right)\left(f\left(x\right)+Bu\right)\\ =Az+J_{h}\left(x\right)Bu, \end{array}$$

which defines an underdetermined system of $d_{z}$ equations with $d_{z}$ unknown transformations and $d_{z}^{2}$ unknown coefficients in matrix A . However, for some special cases, a closed-form solution can be found directly, such as when $d_{x}=d_{z}=1$ . In general, however, equation (2.5) cannot be solved directly.

Alternatively, we propose to solve it by reformulating equation (2.5) into an optimization problem that can be solved using data measured from the system. Specifically, an objective function can be defined as the squared Euclidean norm of the difference between equations (2.3) and (2.5):


$$L\left(x,u;h;A\right)=\|J_{h}\left(x\right)\frac{dx}{dt}-\left[Az+J_{h}\left(x\right)Bu\right]{\|}_{2}^{2},$$

where L∈ℝ and $\|\cdot {\|}_{2}:R^{d_{z}}\to R.$

The unconstrained optimization problem can be defined as finding $h^{⋆}$ and $A^{⋆}$ such that


(2.6) $$h^{⋆},A^{⋆}\in \underset{h\in F,A\in R^{d_{z}\times d_{z}}}{\arg\;\min}L\left(x,u;h;A\right).$$

However, optimization over function spaces as in equation (2.6) is often difficult and intractable. Therefore, an alternative strategy involves parametrizing the space of functions or establishing a set of basis functions from which the broader function space can be derived, as discussed in Sasane [40]. In this article, we adopt this alternative approach. Specifically, we employ a deep neural network to parametrize the function h in equation (2.2):


(2.7) $$z=h^{\theta }\left(x\right),$$

where $\theta \in {\mathbb{R}}^{d_{n}}$ is the weight of neural networks. With A also parameterized by neural networks denoted as $A^{\omega }$ through $\omega \in {\mathbb{R}}^{d_{z}\times d_{z}}$ , the optimization problem defined in equation (2.6) can be reformulated into a manageable form, solely involving the optimization of parameters:


(2.8) $$\theta^{⋆},\omega^{⋆}\in \underset{\theta \in {\mathbb{R}}^{d_{n}},\omega \in {\mathbb{R}}^{d_{z}\times d_{z}}}{\text{arg}\;\text{min}}L(x,u;\theta ,\omega )$$

with


(2.9) $$L\left(x,u;\theta ;\omega \right):=\;\|J_{\theta }\left(x\right)\frac{dx}{dt}-\left[A^{\omega }h^{\theta }\left(x\right)-J_{\theta }\left(x\right)Bu\right]{\|}_{2}^{2},$$

where $J_{\theta }$ is the Jacobian matrix of the deep neural network with regard to the parameter θ .

Note that equation (2.8) is trivially minimized by the solution θ=0 and ω=0 . To avoid such issues, a regularization term was added to the objective function. In essence, the additional term defines a ‘decoder’ network [41] to perform the inverse transformation from the new state z , back to a reconstruction of x , which we denote as $\hat{x}$ with


(2.10) $$\hat{x}={\hat{h}}^{\hat{\theta }}\left(z\right),$$

where $\hat{h}$ is the neural network with weight $\hat{\theta }\in {\mathbb{R}}^{d_{z}\times d_{\hat{n}}}$ . Combining equations (2.7) and (2.10), the decoder’s objective is then defined to minimize


(2.11) $$\hat{L}\left(x;\hat{\theta }\right)=\|x-\hat{x}{\|}_{2}^{2}=\|x-{\hat{h}}^{\hat{\theta }}\left(h^{\theta }\left(x\right)\right){\|}_{2}^{2}.$$

The overall objective function consists of a weighted sum of equations (2.9) and (2.11), which gives rise to the optimization problem:


(2.12) $$\min_{\theta ,\hat{\theta },\omega }L\left(x,u;\theta ;\omega \right)+\alpha \hat{L}\left(x;\hat{\theta }\right),$$

where α defines a scalar weighting factor applied to the decoder term.

The optimization problem can now be solved, provided that data measured from the system are available. In the case of equation (2.12), additional data in the form of the input measurements, u , through time are necessary. This gives the final optimization solved numerically over the data:


(2.13) $$\begin{array}{c} \theta^{⋆},{\hat{\theta }}^{⋆},\omega^{⋆}\in \underset{\theta ,\hat{\theta },\omega }{\arg\;\min}\frac{1}{T}\sum_{t=1}^{T}L^{s}\left(s_{t};\theta ,\omega ,\hat{\theta }\right)\\ L^{s}\left(s_{t};\theta ;\omega ,\hat{\theta }\right):=L\left(s_{t};\theta ,\omega \right)+\alpha \hat{L}\left(x_{t};\hat{\theta }\right), \end{array}$$

where $s_{t}:=(x_{t},u_{t})$ and T is the number of samples in the data and superscript defines the t th sample.

The learned linear dynamics of equation (2.5) can be represented as:


(2.14) $$\frac{dz}{dt}=A^{\omega^{⋆}}z+J_{\theta^{⋆}}\left(x\right)Bu.$$

The optimal control problem can be solved to control nonlinear dynamic systems of the form of equation (2.1), using the learned linear representation of the form of equation (2.14), by solving the Riccati equation for the optimal gain matrix, $K\in {\mathbb{R}}^{d_{u}\times d_{z}}$ , giving the optimal control law:


(2.15) $$u^{⋆}=-Kz,$$

referred to as the linear quadratic regulator [39].

Note that equation (2.14) is linear in z , but not necessarily jointly linear in the inputs and states owing to the Jacobian term, $J_{\theta^{*}}(x)$ , which may be dependent on the nonlinear state x . As discussed in Kaiser *et al.* [30], though the nonlinear state-dependent term does not pose any major issues with regard to control of the nonlinear system or the linear system, in practice, the Jacobian matrix can be ill-conditioned during the initial phase of learning, making equation (2.15) unavailable. In this article, we propose a DRL agent to track the reference signal incurred by equation (2.15) whenever it is available and penalize the DRL agent whenever it is not. We show that the control law learned by this DRL agent is better than the one directly optimize it over a nonlinear system without a mapping.

### 2.2. Reinforcement learning algorithm design

In this section, we introduce the use of a DRL algorithm to explore possible policies that will cover as many scenarios of the nonlinear dynamics with inputs as possible in the case when the optimal control input from the learned linear representation is not available. Meanwhile, such exploration should adhere to constraints that account for the physical and biological limitations inherent to the wound healing system, while ensuring ethical considerations are not compromised.

When the optimal control input is accessible, the DRL algorithm should be able to exploit its acquired knowledge to generate a policy that closely approximates the resulting nonlinear state to the one achieved through control based on the optimal control. The exploration and exploitation of the DRL algorithm do not require knowledge of either nonlinear or linear dynamics, and thus it not only alleviates the burden of mathematical interpretation in real-world treatment scenarios but also significantly expedites the healing process. To realize this, we first formulate the wound healing dynamics as the Markov decision process (MDP) problem and subsequently solve it using the famous Deep Q-learning [42].

We consider a MDP defined by (X,U,P,r,γ) , where X represents the state space, U represents the input/action space, P represents the transition probability matrix, r represents the reward function and γ represents the discount factor. In MDP, an autonomous agent makes sequential discrete-time decisions as time passes. Generally speaking, the MDP problem conforms to the decision-making process of physicians in wound care. Based on the state $x_{t}\in X$ , the agent selects action $u_{t}\in U$ at time t , then it observes the next state $x_{t+1}$ and receives the reward $r(x_{t},u_{t})\in \mathbb{R}$ . To collect more state information in wound management, the agent can perform state observation more frequently, such as a state observation every hour and action selection every 20 min [9,38]. The state $s_{t}$ transits to the next state $x_{t+1}$ following the transition probability matrix $P(x_{t+1}|x_{t},u_{t})$ , which represents the dynamics of the operating environment. The transition probability matrix satisfies the Markovian (or memoryless) property since a transition to the next state $x_{t+1}$ depends only on the current state $x_{t}$ and action $u_{t}$ rather than a historical series of states and actions. The agent learns the optimal policy $\phi^{⋆}:X\to U$ , which maps x∈X to optimal actions u∈U over trial and error interaction with the environment. Nevertheless, the transition probability matrix and the probability distribution of the reward function are generally unknown in reality.

**Deep Q-learning** The goal of an RL agent is to interact with the environment by selecting actions to maximize cumulative future rewards. We make the standard assumption that future rewards are discounted by a factor of γ per time step and define the optimal action-value Q-function as the maximum total discounted expected reward over all possible action sequences $U:=\{u_{t}:t\geq 1\}$ :


$$\begin{array}{c} Q^{⋆}\left(x,u\right)=\underset{U}{max}\sum_{t=0}^{\infty }\gamma^{t}E\left[r\left(x_{t},u_{t}\right)|x_{0}=s,u_{0}=u\right]\\ =\underset{U}{max}\sum_{t=0}^{\infty }\sum_{x^{\prime }\in X}P\left(s^{\prime }|s_{t},u_{t}\right)\left(r\left(x_{t},u_{t}\right)+\gamma \underset{u^{\prime }}{max}Q^{⋆}\left(x^{\prime },u^{\prime }\right)\right), \end{array}$$

with x∈X and u∈U .

Let $P_{u}$ denote the state transition matrix when action u∈U is taken. It is known that the Q-function is the unique solution to the Bellman equation [43]:


$$Q^{⋆}\left(x,u\right)=r\left(x,u\right)+\gamma \sum_{x^{\prime }\in X}P_{u}\left(x,x^{\prime }\right){\underset{\_}{Q}}^{⋆}\left(x^{\prime }\right),$$

where $\underline{Q}(x):=\underset{u\in U}{\max}Q(x,u)$ for any function Q:X×U→ℝ .

Consider a parametrized family of approximations $\left\{Q^{\vartheta }:\vartheta \in {\mathbb{R}}^{d}\right\}$ , wherein $Q^{\vartheta }:X\times U\to \mathbb{R}$ and ϑ may represent the weights from deep neural networks. The associated family of policies is defined as


(2.16) $$\phi^{\vartheta }\left(x\right)\in \underset{u\in U}{\arg\;\max}Q^{\vartheta }\left(x,u\right),\;\;\;\;x\in X.$$

The goal of the Deep Q-network (DQN) algorithm is to find $\vartheta^{⋆}$ such that the mean square Bellman error is minimized:


(2.17) $$\vartheta^{⋆}\in \underset{\vartheta \in R^{d}}{\arg\;\min}E\left[\|D_{t+1}\left(\vartheta \right){\|}_{2}^{2}\right],$$

where $D_{t+1}(\vartheta ):=r(x_{t},u_{t})+\gamma {\underline{Q}}^{\vartheta }(x_{t+1})-Q^{\vartheta }(x_{t},u_{t})$ , and the expectation is in a steady state.

To balance the trade-off between exploration and exploitation, we adopt the ε -greedy policy approach, where ε follows the following updating rule:


(2.18) $$\varepsilon_{t+1}=\max\left(\varepsilon_{\min},\nu \varepsilon_{t}\right),$$

with $\varepsilon_{\text{min}}$ the minimum value that ε can achieve and 0<ν<1 the decay rate.

Initially, $\varepsilon_{0}$ is set to a value close to 1, such as 0.99. This initial value encourages a higher probability of random selection of action $u_{t}\in U(x_{t})$ with U(x)⊆U constrained by the current state. This choice aligns with the early stages of training when both the transformation $h^{\theta }$ and the DRL agent are still in the learning process and are not yet well-versed. As they are continuously updated through trajectories collected from real-world experiments or simulated wound dynamics, they gain more confidence in the learned linear representation and nonlinear dynamics. Thus, we should gradually decrease ε and guide the policy towards more deterministic actions.

The optimization problems of equations (2.12) and (2.17) are then solved iteratively using data collected by interacting with some wound dynamics as per Algorithm 1.

The steps for obtaining optimal solutions are summarized as follows:

**Step 1: Hyperparameter setting** The weighting faction α , applied to the decoder term in equation (2.12), is initialized with value $\alpha^{{}^{\circ}}$ . The exploration rate ε is set to value $\varepsilon^{{}^{\circ}}$ .

**Step 2: Deep neural network initialization** The initial weights of the deep Q networks $\vartheta^{{}^{\circ}}$ , transformer $\theta^{{}^{\circ}}$ , decoder ${\hat{\theta }}^{{}^{\circ}}$ and $A^{\omega }$ matrix $\omega^{{}^{\circ}}$ are randomly selected by the Kaiming uniform method [44,45].

**Step 3: Learning through data** A while loop is initiated until the termination criteria are satisfied. That is, either the optimal parameters between iterations are similar, where similarity is measured with a Euclidean distance metric, or the maximum number of iterations is exceeded. Within this while loop, we have another while loop that keeps interacting with the nonlinear wound dynamics using control inputs either obtained from equation (2.15) or the randomly selected one based on some constrained input space U(x) . This interaction will stop until the wound has healed. All the data during the interaction will be stored for optimizing the parameters. The optimization problems considered in this article were solved using the Adam optimizer [46].

**Step 4: Return** The optimal of parameters, $\vartheta^{⋆}$ , $\theta^{⋆}$ , $\hat{\theta^{⋆}}$ , and $\omega^{⋆}$ will be obtained by using the Polyak–Ruppert averaging method defined in equation (3.4).


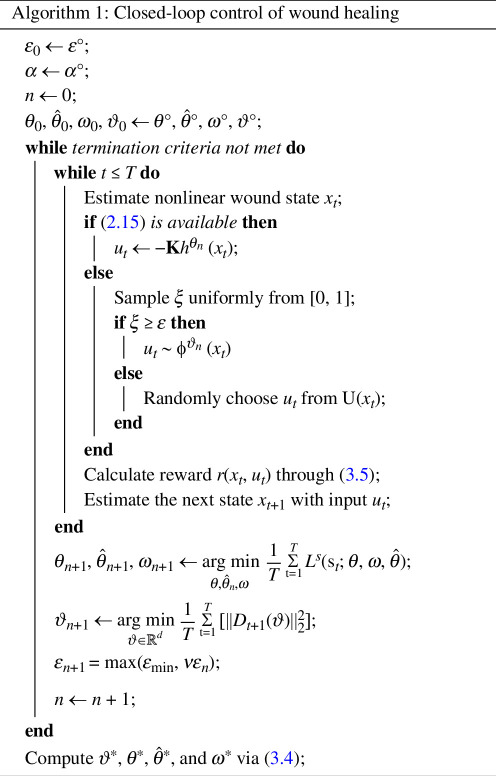


## 3. Experiment results and discussion

We evaluate the proposed algorithm by applying it to a nonlinear model, as introduced in Zlobina *et al.* [9]. This nonlinear model addresses wound healing by encompassing five key variables: the quantity of debris a , M1 macrophage $m_{1}$ , M2 macrophage $m_{2}$ , temporal tissue c and new tissue n . We assume in equations (3.1*a*)–(3.1*e*) that the control factor u only impacts the rate of transitions from $m_{1}$ to $m_{2}$ . Consider a circular wound with radius R and denote $x:={\left[a,m_{1},m_{2},c,n\right]}^{⊺}$ . Each element in x evolves nonlinearly in both the spatial dimension $0\leq \tilde{r}\leq R$ and temporal dimension 0≤t :


(3.1*a*) $$\overset{˙}{a}=-am_{1},$$

(3.1*b*) $$\dot{m_{1}}=\beta a-\overset{˙}{a}-\rho \frac{m_{1}^{q}}{k^{q}+m_{1}^{q}}-\gamma_{1}m_{1}+\overset{˜}{D}F\left(m_{1}\right)-um_{1},$$

(3.1*c*) $$\dot{m_{2}}=\rho \frac{m_{1}^{q}}{k^{q}+m_{1}^{q}}-\gamma_{2}m_{2}+\overset{˜}{D}F\left(m_{2}\right)+um_{1},$$

(3.1*d*) $$\overset{˙}{c}=m_{2}-\mu c,$$

(3.1*e*) $$\overset{˙}{n}=c\left[\overset{˜}{\alpha }n\left(1-n\right)+{\overset{˜}{D}}_{n}F\left(n\right)\right],$$

where $F(x):=\frac{1}{\tilde{r}}\frac{\partial x}{\partial \tilde{r}}+\frac{\partial^{2}x}{\partial {\tilde{r}}^{2}}$ for any variable x , the wound radius $\tilde{r}$ is directed from the wound centre to the wound edge and u~ϕ with $\phi :{\mathbb{R}}^{+}\times {\mathbb{R}}^{+}\to {\mathbb{R}}^{+}$ a function of space and time that modifies the polarization of $m_{1}$ to $m_{2}$ and affects the rate of the generation of new tissues. Note that equations (3.1*a*)–(3.1*e*) can be written in the form of equation (2.1) with the matrix B defined as


$$B:=\left[\begin{array}{ccccc} 0 & 0 & 0 & 0 & 0\\ 0 & -1 & 0 & 0 & 0\\ 0 & 0 & 1 & 0 & 0\\ 0 & 0 & 0 & 0 & 0\\ 0 & 0 & 0 & 0 & 0 \end{array}\right],$$

$u:={\left[0,u,u,0,0\right]}^{⊺}$ and f capturing the first and second derivatives of variables. We assume that f is unknown to the algorithms and x is measurable through some sensor attached to the wound.

We define the wound size at time t as the smallest radius where the new tissue reaches a value of σ :


$$s\left(t\right)=\underset{\overset{˜}{r}}{min}\;n\left(t,\overset{˜}{r}\right)\geq \sigma .$$

The wound healing time is defined as the time from injury ( t=0 ) to the moment when the wound radius is zero:


(3.2) $$\tau =\underset{t\geq 0}{min}\;s\left(t\right)=0.$$

The goal is to find an actuation function ϕ such that τ is minimized. Nevertheless, solving for the optimal $\phi^{⋆}$ directly from equations (3.1*a*)–(3.1*e*) is often difficult, particularly when involving second-order derivatives. In the previous work [9], a brute-force search (BFS) method was used. While effective, this approach was not only time-intensive but also heavily reliant on prior knowledge of the structures of the actuation function, which may not cover the true optimal solution. Consequently, there is still untapped potential for expediting wound healing by employing more advanced approaches.

In this section, we first use the method proposed in §2 to learn a linear representation of the system (3.1*a*)–(3.1*e*) without any actuation ( u=0 ). Our primary motivation for this is twofold: firstly, to demonstrate the capability of our proposed method in acquiring a meaningful linear representation of the nonlinear system; and secondly, to unveil biologically interpretable insights into the variables embedded within the linear model. We then consider this learned linear representation as prior knowledge to find a linear representation for the nonlinear model with control inputs guided by a DRL agent. The experimental results show that the learned policy $\phi^{⋆}$ is capable of reducing the healing time by 45.56% compared to that without any actuation and 37% compared to that with the method employed in Zlobina *et al.* [9].

We summarize all parameter setups during each experiment in table 1.

**Table 1.** The values of parameters used in experiments.

parameter	R	L	T	β	ρ	κ	q	$\gamma_{1}$	$\gamma_{2}$	μ	D	${\tilde{D}}_{n}$	$\tilde{\alpha }$	γ	$\varepsilon_{\text{min}}$	σ	ν
value	3 mm	0.03 mm	1/3 day	1	0.1	0.05	5	0.1	0.1	0.2	0.32	$3\times 10^{-4}$	1.8	0.995	0.01	0.95	0.99

### 3.1. Learning the linear representation for a nonlinear model without control input

We assume that there are four variables in the linear representation, and each corresponds to the probability of each stage of wound healing: haemostasis $P_{h}$ , inflammation $P_{i}$ , proliferation $P_{p}$ and maturation $P_{m}$ . As time goes on, the probability of each stage changes: the wound is initially in the hemostasis stage with probability one and experiences a continuous transition from stage to stage.

During the experiment, we found that the learned linear representation is not unique. This is owing to the fact that we are mapping a nonlinear dynamic to a lower-dimensional linear model. To drive the uniqueness of the output of such mapping, we introduce a four-state ODE model:


(3.3*a*) $$\frac{dz}{dt}=Az+Wu,\;\;\;\;z={\left[P_{h},P_{i},P_{p},P_{m}\right]}^{⊺}\in R^{4},$$

(3.3*b*) $$A=\left[\begin{array}{cccc} -k_{h}^{\text{nat}} & 0 & 0 & 0\\ k_{h}^{\text{nat}} & -k_{i}^{\text{nat}} & 0 & 0\\ 0 & k_{i}^{\text{nat}} & -k_{p}^{\text{nat}} & 0\\ 0 & 0 & k_{p}^{\text{nat}} & 0 \end{array}\right],$$

where $k_{h}^{\text{nat}}$ , $k_{i}^{\text{nat}}$ and $k_{p}^{\text{nat}}$ are the constants that control the velocities of transitions without any treatment for the wound, and the matrix $W\in {\mathbb{R}}^{d_{z}\times d_{u}}$ needs to be learned to capture the input effect in the linear model from the nonlinear dynamics.

**Data preparation** In order to find the linear representation for the nonlinear model without any control inputs, we first solve equations (3.1*a*)–(3.1*e*) numerically by constructing 500 ordinary differential equations on a uniform mesh consisting of 100 spatial cells similar to Zlobina *et al.* [9]. The temporal domain spans from t∈[0,20] with a sampling interval of 0.5. This yields a dataset composed of 121 data points, each characterized by 500 features.

To enlarge the dataset and promote robustness in our results, we introduce additional variability by adding i.i.d. noise. This noise is sampled from a uniform distribution in the range of ${\left[-0.1,0.1\right]}^{500}$ and is incorporated into the original dataset. This data augmentation increases the size of the dataset to 12 100 data points, which serves as the training data for solving the optimization problem (equation (2.12)).

The neural network that approximates the function $h^{\theta }$ has three fully connected layers with the Softmax function as the output layer. The network approximating function ${\hat{h}}^{\hat{\theta }}$ has a similar structure but with the output layer replaced by a Sigmoid activation function. The reason for this replacement is that the five variables in the nonlinear dynamics are not necessarily the probabilities, but the four variables in the linear model are. We constrained the parameters for the $A^{\omega }$ matrix to have the same structure as that in equation (3.3*b*).

**Figure 2.**
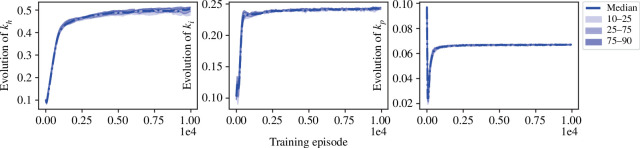
Values of $k_{h}^{\text{nat}}$ , $k_{i}^{\text{nat}}$ and $k_{p}^{\text{nat}}$ during learning, shown by percentile.

We conduct 100 independent runs of learning those approximations, with parameters in the neural networks randomly initialized by the Kaiming uniform method [44,45], and obtain the learning curves of $k_{h}^{\text{nat}}$ , $k_{i}^{\text{nat}}$ and $k_{p}^{\text{nat}}$ shown in figure 2, where all the three parameters converge after around $6\times 10^{3}$ epochs. To get a better estimation of these values, we conduct Polyak–Ruppert averaging [47]:


(3.4) $$\omega_{N}^{⋆}:=\frac{1}{N-N_{0}}\sum_{n=N_{0}}^{N}\omega_{t},$$

where N denotes the total number of updates in the parameters, and the interval $\left[0,N_{0}\right]$ with $N_{0}<N$ is known as the *burn-in* period; estimates from this period are abandoned to reduce the impact of transients in early stages of the training. In this article, we choose $N_{0}=80\%\;N$ and obtain


$$A^{\omega_{T}^{⋆}}=\left[\begin{array}{cccc} -0.495 & 0 & 0 & 0\\ 0.495 & -0.247 & 0 & 0\\ 0 & 0.247 & -0.068 & 0\\ 0 & 0 & 0.068 & 0 \end{array}\right].$$

**Figure 3.**
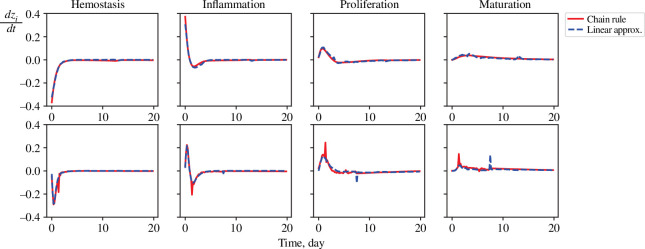
Results of the optimization showing a comparison between the exact time derivative calculated through the chain rule (red curve), i.e. $\frac{dz}{dt}=J_{\theta^{⋆}}\left(x\right)\frac{dx}{dt}$ , and their linear approximation (blue dashed curve), i.e. $\frac{dz}{dt}=A^{\omega^{⋆}}z+J_{\theta^{⋆}}\left(x\right)Bu$ , with u=0 in the first row and $u∼\phi^{⋆}$ in the second row.

Figure 3 shows the optimization result for the original trajectory given by equations (3.1*a*)–(3.1*e*). The solid red curves represent the exact time derivative calculated from the chain rule, i.e. $\frac{dz}{dt}=J_{\theta^{⋆}}\left(x\right)\frac{dx}{dt}$ . In contrast, the blue dashed curves are the results of the linear approximation, i.e. $\frac{dz}{dt}=A^{\omega^{⋆}}z$ . These plots show that a linear model has been successfully identified with all the ODEs converging to zero.

**Figure 4.**
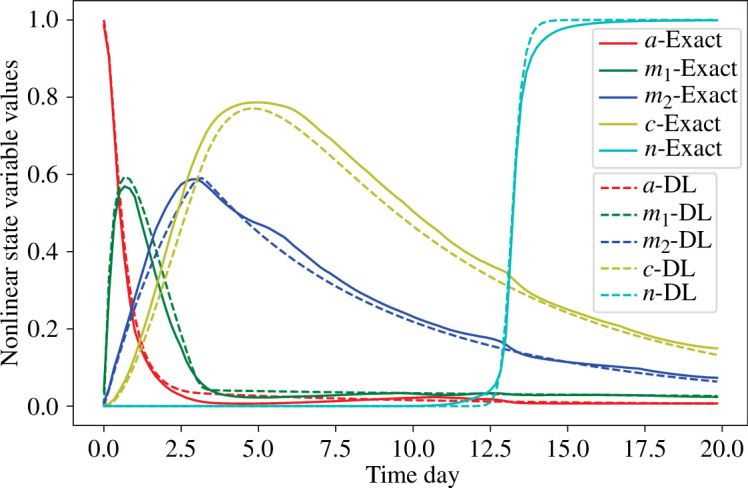
Results of all time-dependent variables in the nonlinear wound healing model (solid curves) and its deep learning decoder approximation (dashed curves) at wound centre ( $\tilde{r}=0$ mm).

In figure 4, it is demonstrated that the decoder ${\hat{h}}^{{\hat{\theta }}^{⋆}}$ has effectively mapped the linear variables into the variables of the nonlinear model, underscoring the accuracy and reliability of this mapping process.

**Figure 5.**
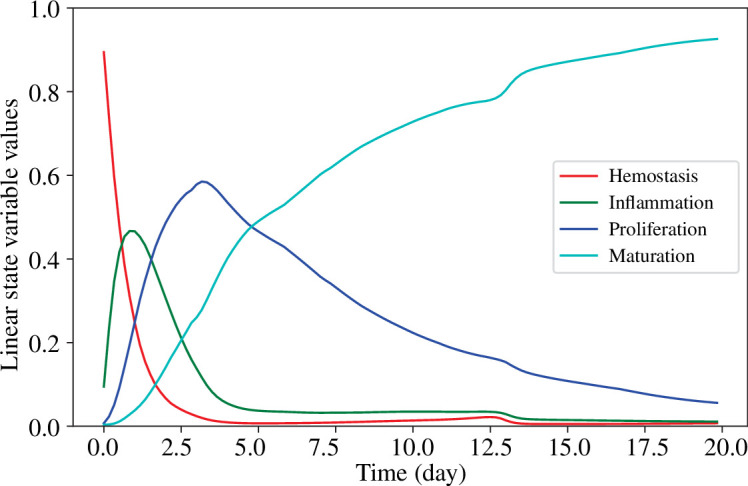
Results of time dependence of all variables in the linear representation.

Plugging matrix $A^{\omega_{T}^{⋆}}$ into equation (3.3*a*), and solving it numerically over a time span from t∈[0,20] , with a sampling interval of 0.5 and u=0 , we derive a trajectory of the four variables shown in figure 5. Compared to figure 4, it can be seen that the trajectories of haemostasis, inflammation and proliferation evolve similarly to those of debris, M1 macrophage and M2 macrophage.

### 3.2. Learning the linear representation for a nonlinear model with deep reinforcement learning control inputs

Subsequently, we proceed to conduct experiments towards acquiring a linear representation of the nonlinear model (equations (3.1*a*)–(3.1*e*)) when it involves control inputs ( u≠0 ) through the DeepMapper. Meanwhile, we would like to simultaneously train a DRL agent to learn an optimal treatment strategy denoted as $\phi^{⋆}$ that minimizes the healing time defined in equation (3.2), which will, in turn, refine the DeepMapper.

In the RL algorithm introduced in §2.2, the Q-network is constructed to have four fully connected layers, and a rectified linear unit (ReLU) activation function follows each layer. The output layer is a Softmax activation function to output the probabilities of each input u∈U .

Note that we divide the wound into 100 regions with wound radius evenly spaced along the direction of $\tilde{r}$ , and $u\in {\mathbb{R}}^{100}$ is a vector with each element indicating the amount of actuation at different radiuses of the wound ranging from [0,R] mm. Each element of u takes values from {0.1n:0≤n≤10,n∈z} . This will result in an input space $U\in {\mathbb{R}}^{10\times 100}$ .

We took the learned models in §3.1 as a prior for the models with control and updated the Q-network as well as $h^{\theta }$ and ${\hat{h}}^{\hat{\theta }}$ in an online learning way. For each state $x_{t}$ at time t , the input $u_{t}$ to the nonlinear model (equations (3.1*a*)–(3.1*e*)) is obtained by either equation (2.15) when the solution to the Riccati equation is available or uniformly sampling it from $U(x_{t})$ . Note that $U(x_{t})$ denotes an input space constrained by $x_{t}$ , so the sampled input will be restricted to the bounds of the wound’s biological and physical dynamics.

The reward at time t is defined as


(3.5) $$r\left(x_{t},u_{t}\right)=\left\{ \begin{array}{cc} e^{-\|{\hat{x}}_{t}^{⋆}-x_{t}\|}-1, & \text{if (2}\text{.15) is available}\\ -2, & \text{otherwise}, \end{array}\right.$$

where $x_{t}$ is the state from the nonlinear dynamics (3.1*a*)–(3.1*e*) with control input $u_{t}$ , ${\hat{x}}_{t}^{⋆}$ is the target state decoded by ${\hat{h}}^{\hat{\theta }}$ from the linear state $z_{t}^{⋆}$ with control input $u_{t}$ obtained by equation (2.15). If such target state ${\hat{x}}_{t}^{⋆}$ is unavailable, a much smaller reward, i.e. −2, is assigned, which will drive the DRL agent to learn a policy to stabilize the linear representation and track the optimal trajectory with optimal input for this linear system.

One may consider the use of the target state for the DRL agent to track is unnecessary. Instead, a more intuitive definition of reward function could be


(3.6) $$r^{{\;}^{\circ }}\left(x_{t},u_{t}\right)=-1\left\{n_{t}\leq 0.95\right\}.$$

We then have two datasets for training the DRL agent:


$$\begin{array}{c} M=\left\{x_{t},u_{t},r\left(x_{t},u_{t}\right),x_{t+1}:t\geq 0\right)\text{and}\\ \overset{˜}{M}=\left\{x_{t},u_{t},r^{{\;}^{\circ }}\left(x_{t},u_{t}\right),x_{t+1}:t\geq 0\right\}. \end{array}$$

We denote the resulting treatment policies as $\phi^{\vartheta^{⋆}}$ and ${\tilde{\phi }}^{\vartheta^{⋆}}$ respectively.

Using $\overset{˜}{M}$ will directly minimize the wound’s healing time based solely on the feedback from the nonlinear dynamics without tracking any target state as discussed in Lewis *et al.* [48]. However, we show in the experiment that ${\tilde{\phi }}^{\vartheta^{⋆}}$ is not a safe and economical treatment policy compared with $\phi^{\vartheta^{⋆}}$ .

**Figure 6.**
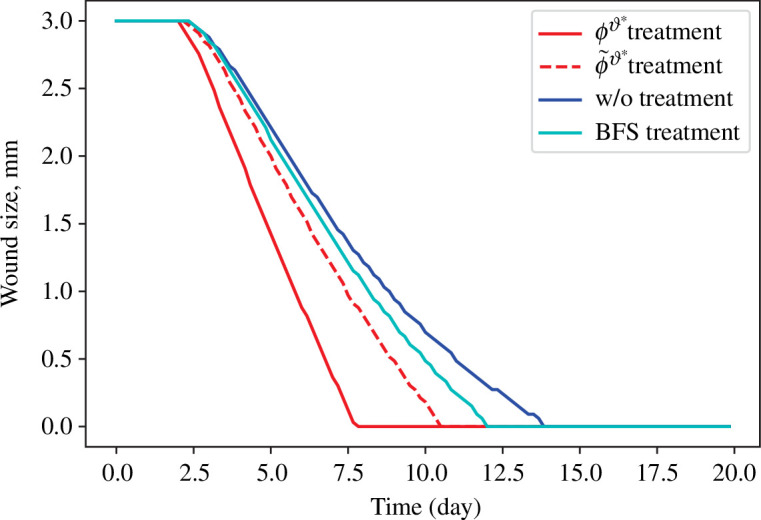
Results of wound size versus time: wound healing time is 7.53 days using $\phi^{\vartheta^{⋆}}$ treatment, 10.67 days with ${\tilde{\phi }}^{\vartheta^{⋆}}$ treatment, 13.83 days without any treatment and 11.33 days with BFS treatment from Zlobina *et al.* [9].

The progressions of wound healing in terms of wound size are shown in figure 6, where we also compared the performances of the treatment strategy given by Zlobina *et al.* [9]. It can be seen from figure 6 that the treatment strategy $\phi^{\vartheta^{⋆}}$ reduces the healing time the most, around 45.56% time of reduction compared to the one without any treatment and 33.54% time of reduction compared to the one with treatment by BFS [9].

**Figure 7.**
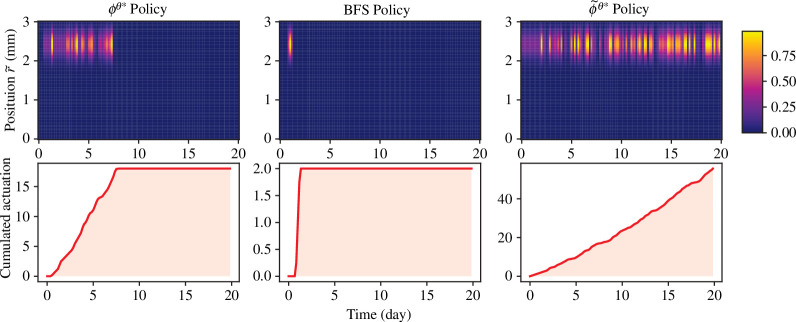
Different policies of wound treatment. The first row gives plots of spatial–temporal actuation given by policies $\phi^{\vartheta^{⋆}}$ , BFS and ${\tilde{\phi }}^{\vartheta^{⋆}}$ . The second row gives the cumulated actuation over time.

The strategy coming from equation (3.5) also provides a safety advantage over the one from equation (3.6) as can be seen from figure 7. As is discovered in Zlobina *et al.* [9], it would be dangerous to apply any treatment at the early stage of the wound, which corresponds to zero actuation indicated by $\phi^{\vartheta^{⋆}}$ and BFS policy shown in figure 7. However, such avoidance in the danger zone is not captured by ${\tilde{\phi }}^{\vartheta^{⋆}}$ , as is shown in the third column plot of figure 7. Compared with the plots of $\phi^{\vartheta^{⋆}}$ and ${\tilde{\phi }}^{\vartheta^{⋆}}$ , we can also observe that $\phi^{\vartheta^{⋆}}$ will stop actuation when the wound has healed, while ${\tilde{\phi }}^{\vartheta^{⋆}}$ keeps actuating. This indicates that the proposed closed-loop control framework is also superior in giving treatment at lower doses.

## 4. Discussion

New biotechnologies have introduced a multitude of sensors for various biological systems, addressing a growing need in medicine to integrate these sensors into closed-loop control systems. However, the complexity of biological processes presents a challenge in formulating accurate mathematical models; thus, there is a demand for control algorithms that do not rely on precise models. While sensors provide valuable insight, their measurements only partially capture the dynamics of real biological systems.

Wound healing serves as an example of a nonlinear process with diverse roles played by different cell types across various stages. In our study, we operate under the assumption that a sensor reflecting wound stages is available [38], and it provides information that can be easily approximated by a linear system of ODEs.

However, owing to discrepancies between measurements and the actual biological components involved in wound healing, linear systems fail to align perfectly with the underlying nonlinear processes.

Nevertheless, we demonstrate the feasibility of monitoring and controlling nonlinear systems through observations derived from linear approximations. We assert that this approach holds promise for a broad spectrum of nonlinear biological processes for which sensors have been developed, but accurate mathematical modelling remains difficult.

Finally, it is worth noting that there remain many other more advanced DRL algorithms that can further improve the control strategy and sample efficiency of the proposed algorithm. For exmaple, when it comes to large state and action spaces, DQN will take much longer time to learn an optimal control strategy and can often fall into local minima. In appendix A, we replaced it with Advantage Actor-Critic (A2C) [49] and showed that it only took around 200 episodes for the proposed algorithm to find a similar treatment strategy and outperformed the one from directly optimizing A2C over the nonlinear system. As our main goal is to propose an adaptive learning structure on how to combine deep learning, optimal control and DRL for accelerating wound healing, we would like to design a better DRL algorithm for future studies.

## 5. Conclusion

In this article, we propose an adaptive closed-loop control framework for a nonlinear dynamical system. The controller integrates deep learning, optimal control and RL, aiming to accelerate nonlinear biological processes such as wound healing without the need for mathematical modelling. We have demonstrated that the proposed method not only significantly improves wound healing time but also addresses safety concerns and reduces drug usage.

Further development of the controller with more advanced DLR algorithms, as well as its implementation in *in vivo* experiments, will ultimately lead to significant improvements in wound care and broader medical domains leveraging intelligent control algorithms.

## Figures and Tables

**Figure 8.**
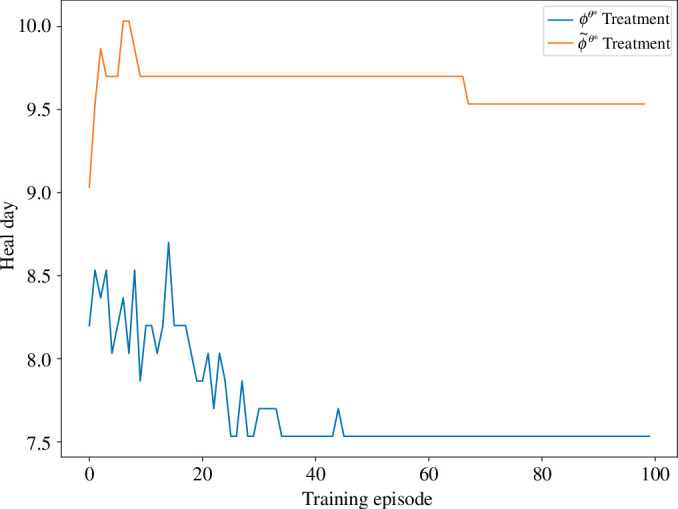
Comparison of expected heal days using different treatment strategies. The DRL algorithm used in both strategies is the A2C.

## Data Availability

Data and relevant code for this research work are stored in GitHub [50] and have been archived within the Zenodo repository [51].

## Appendix. A

We further explored replacing the DQN algorithm in the proposed algorithm with the Advantage Actor-Critic algorithm (A2C) [49] and comparing it with directly finding control policies with A2C through interactions with nonlinear dynamics. As a result, we have two types of control policy $\phi^{\theta^{*}}$ obtained from optimizing A2C agent using M and ${\tilde{\phi }}^{\theta^{*}}$ obtained from optimizing A2C agent using $\overset{˜}{M}$ . Definitions of M and $\tilde{M}$ can be found in §3.2.

Note that when making the comparison, we keep the neural network structures, optimizers and hyperparameters, such as learning rate, discount factor, etc., all the same.

As can be seen from figure 8, while A2C is still learning the strategy when it is directly optimized over the nonlinear system, our proposed algorithm has converged to the treatment strategy that results in much shorter healing days. This further reveals our algorithm’s advantages in sample efficiency.
